# Recent advances and trends of trichloroethylene biodegradation: A critical review

**DOI:** 10.3389/fmicb.2022.1053169

**Published:** 2022-12-22

**Authors:** Zhineng Wu, Quanli Man, Hanyu Niu, Honghong Lyu, Haokun Song, Rongji Li, Gengbo Ren, Fujie Zhu, Chu Peng, Benhang Li, Xiaodong Ma

**Affiliations:** ^1^School of Energy and Environmental Engineering, Hebei University of Technology, Tianjin, China; ^2^MOE Key Laboratory of Pollution Processes and Environmental Criteria, College of Environmental Science and Engineering, Nankai University, Tianjin, China

**Keywords:** anaerobic, aerobic, biodegradation, mechanism, trichloroethylene

## Abstract

Trichloroethylene (TCE) is a ubiquitous chlorinated aliphatic hydrocarbon (CAH) in the environment, which is a Group 1 carcinogen with negative impacts on human health and ecosystems. Based on a series of recent advances, the environmental behavior and biodegradation process on TCE biodegradation need to be reviewed systematically. Four main biodegradation processes leading to TCE biodegradation by isolated bacteria and mixed cultures are anaerobic reductive dechlorination, anaerobic cometabolic reductive dichlorination, aerobic co-metabolism, and aerobic direct oxidation. More attention has been paid to the aerobic co-metabolism of TCE. Laboratory and field studies have demonstrated that bacterial isolates or mixed cultures containing *Dehalococcoides* or *Dehalogenimonas* can catalyze reductive dechlorination of TCE to ethene. The mechanisms, pathways, and enzymes of TCE biodegradation were reviewed, and the factors affecting the biodegradation process were discussed. Besides, the research progress on material-mediated enhanced biodegradation technologies of TCE through the combination of zero-valent iron (ZVI) or biochar with microorganisms was introduced. Furthermore, we reviewed the current research on TCE biodegradation in field applications, and finally provided the development prospects of TCE biodegradation based on the existing challenges. We hope that this review will provide guidance and specific recommendations for future studies on CAHs biodegradation in laboratory and field applications.

**Figure:**
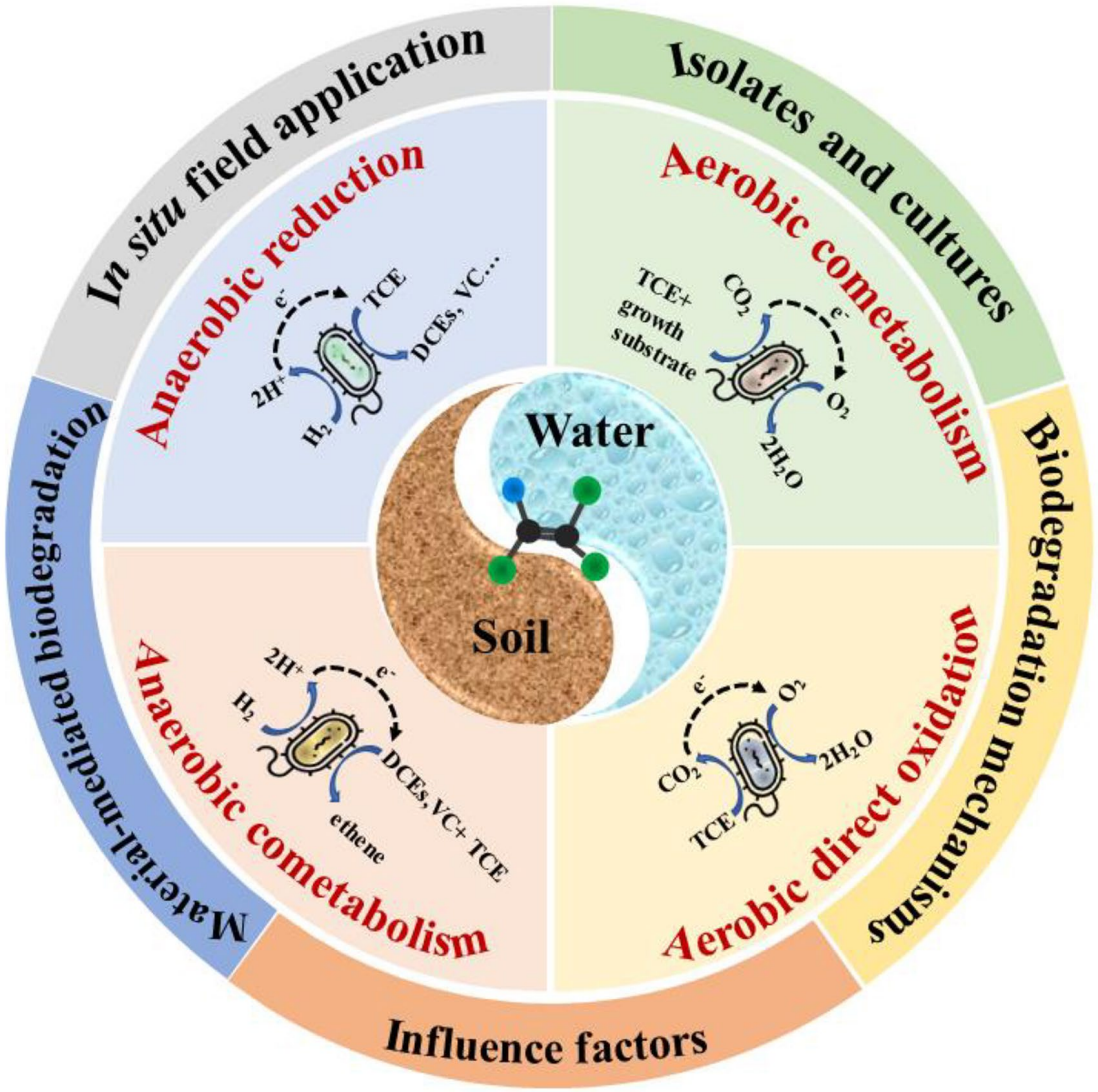
GRAPHICAL ABSTRACT

## 1. Introduction

Trichloroethylene (TCE) is a chlorinated aliphatic hydrocarbon (CAH) that belongs to a class of chlorinated organic solvents (Honetschlägerová et al., 2019). The physicochemical properties of TCE are listed in Table 1 (Pant and Pant, 2010). In 2011, the global consumption of TCE has risen sharply to 428,000 tons (Baskaran and Rajamanickam, 2019), and TCE production was about 250,000 tons in China (Huang et al., 2014). Annual production of TCE in the United States (United States) is about 130,000 metric tons. TCE is used widely in various commercial and industrial applications (Shukla et al., 2014) and in many household products (Suttinun et al., 2013). Due to the inappropriate handling, storage, disposal, and accidental release, TCE has been detected ubiquitously in soil and groundwater in many countries worldwide (Xing et al., 2022; Yamazaki et al., 2022), including the United States (Steffan et al., 1999; Lee et al., 2012; de Guzman et al., 2018), Canada (Perez-de-Mora et al., 2014), France (Dugat-Bony et al., 2012; Walaszek et al., 2021), and China (Kuo et al., 2004; Kao et al., 2016). TCE contamination is known to account for 22% of soil and groundwater at superfund sites in the USA (USEPA, 2013), and was found in 57% of National Priorities List sites in 2015 (ATSDR, 2011; de Guzman et al., 2018).

**Table 1:** Physicochemical properties of TCE.

Properties	Value
Chemical formula	C_2_HCl_3_
Molecular weight	131.4 g/mole
Boiling point	87.2°C
Melting point	−84.7°C
Density (g/cm^3^) at 20°C	1.4642
Solubility in water at 25°C	1.280 g/L
Vapor pressure at 25°C	69.8 mmHG
Air concentration conversion	1 ppb = 5.38 μg/m^3^
Solubility in organic solvents	Highly soluble in ethanol or chloroform
Log_10_ octanol/water partition coefficient (log K_ow_)	2.36
Henry’s law constant (dimension less)	0.397
Log_10_ adsorption coefficient (log K_ac_)	2.6–2.7
Refractive index at 25°C (n_o_)	1.48
Odor threshold	3.9

**Figure 1:**
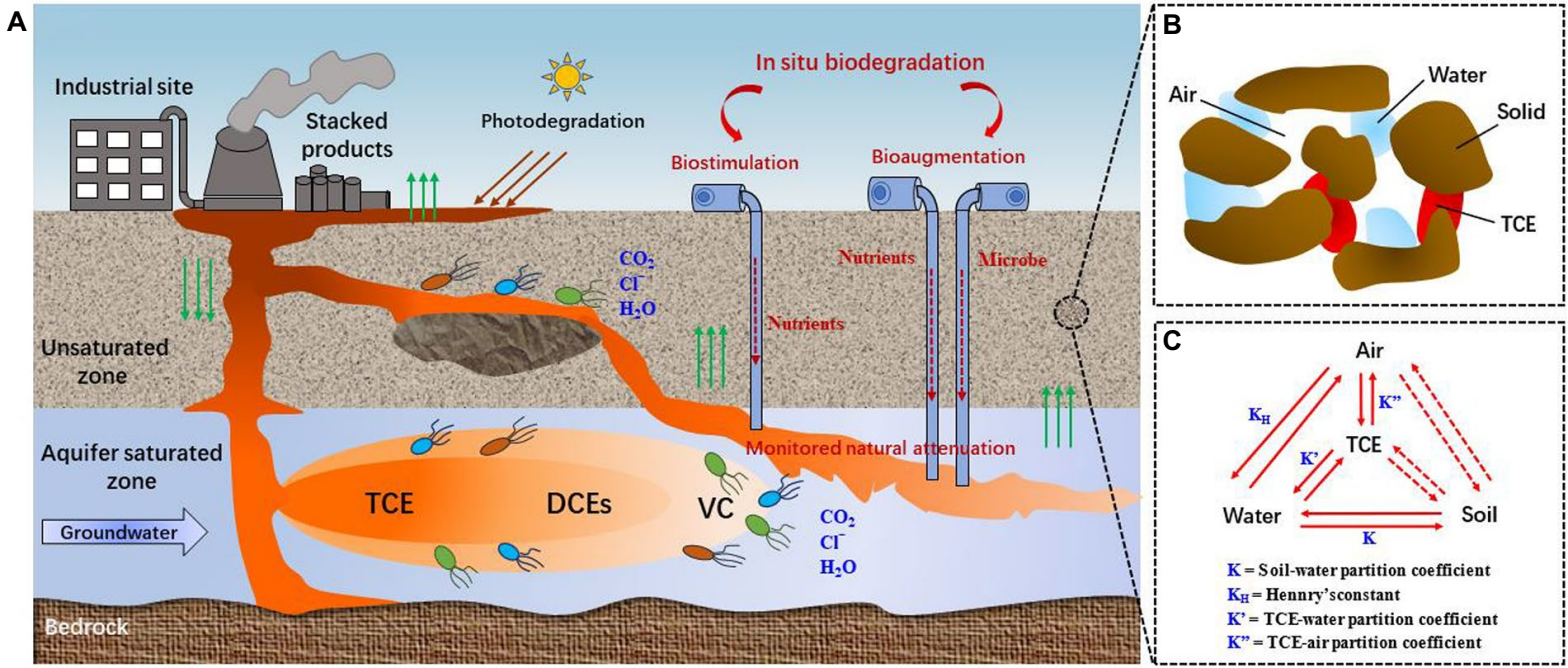
Migration and fate of TCE underground. **(A)** Conceptual model of the transportation of TCE underground [reproduced from (Xing et al., 2022)]; **(B)** Interactions of different phases of TCE; **(C)** Phase distribution of TCE in unsaturated zone.

The migration and fate of TCE in environment depend on the physicochemical properties of TCE and the hydrogeological characteristics of sites (Honetschlägerová et al., 2019). TCE has high molecular weight and is labeled as dense non-aqueous phase liquid (DNAPL), it tends to migrate from the unsaturated zone into the underlying aquifer (Figure 1A). When TCE reaches the clay layer through the subsoil, some TCE may accumulated by subsurface solids (Figure 1B), or is transported through the clay layer (Honetschlägerová et al., 2019). The phases distribution of TCE in unsaturated zone is presented in Figure 1C. Furthermore, TCE may adsorbed by organic and mineral components in groundwater matrices, such as sediments and rocks, which may prevent partial TCE from migrating (Pant and Pant, 2010). TCE residues can be a major source of long-term contamination. As the groundwater flows, so does the contaminated water, creating a pollution plume. At the same time, TCE slowly dissolves into the groundwater, reaching the dissolution limit (Honetschlägerová et al., 2019). In this case, not only TCE is presented in the plume but also reduced products such as cis-1,2-dichloroethylene (cis-DCE), trans-1,2-dichloroethene (trans-DCE), 1,1-dichloroethene (DCE), and vinyl chloride (VC) exist (Honetschlägerová et al., 2019).

TCE is a ubiquitous environmental contaminant, which has adverse effects on both ecosystem and human health (Tachachartvanich et al., 2018; Xiao et al., 2020). As a Group 1 carcinogen, TCE may increase the cancer risk of the kidney, cervix, liver and biliary passages, as well as non-Hodgkin lymphoma and esophageal adenocarcinoma (Johnson et al., 1998; Wartenberg et al., 2000; Hansen et al., 2013; Vlaanderen et al., 2013; Siggins et al., 2021). In addition, TCE has been reported to cause endocrine disrupting effects (Tachachartvanich et al., 2018). Concerns about the toxicity of TCE have driven its control. TCE has been listed as a priority pollutant by the Ministry of Ecology and Environment of China, the United States Environmental Protection Agency (USEPA), and the European Commission (Liu et al., 2021). The USEPA has set an upper limit for TCE concentration in drinking water at 5 μg/L (USEPA, 2009). In China, the Grade V criterion of TCE is 210 μg/L according to the National Standard for Groundwater Quality (GB/T14848-2017). The soil pollution control standard of TCE is 20 mg/kg according to the Soil Environmental Quality Risk Control Standard for soil contamination of development land (GB36600-2018).

Over the past 50 years, human efforts have been made to remediate TCE pollution (Suttinun et al., 2013). Biodegradation of TCE has received extensive attention from researchers due to its cost-effectiveness, environmental friendliness, and sustainability (Shukla et al., 2014; Wang et al., 2022). Biodegradation utilizes the catabolic capabilities of microorganisms with specific enzymatic activities to degrade TCE (Shukla et al., 2014). So far, many advances have been made in the field of biodegradation of chlorinated compounds (Bhatt et al., 2007; Dolinova et al., 2017), such as aerobic co-metabolism strategies of chlorinated solvents (Semprini, 1997) and aliphatic organochlorine degradation in subsurface environments (Koenig et al., 2015). However, only four reviews were published on TCE biodegradation in 1995–2022, including biodegradation of TCE (Pant and Pant, 2010; Shukla et al., 2014), molecular and cellular fundamentals of TCE aerobic co-metabolism (Arp et al., 2001), and co-metabolism of TCE (Suttinun et al., 2013). There has been a gradual increase in the number of studies on TCE biodegradation in recent years (Islam et al., 2021; Liu et al., 2021; Qiu et al., 2022; Underwood et al., 2022), but no recent review on this topic has been published since 2014. Therefore, the environmental behavior and biodegradation process of TCE need to be reviewed systematically.

Here, an effort has been made to shed light on recent studies on TCE biodegradation. The specific objectives of this review are: (1) to review four main biodegradation processes that lead to TCE biodegradation by bacterial isolates and mixed culture: anaerobic reductive dechlorination, anaerobic cometabolic reductive dichlorination, aerobic co-metabolism, and aerobic direct oxidation; (2) to discuss the mechanisms, pathways, enzymes, and influence factors involved in TCE biodegradation; (3) to summarize the material-mediated enhanced TCE biodegradation technology; (4) to review research progress of *in situ* biodegradation of TCE, and to propose the future perspectives of TCE biodegradation.

## 2. Effects of TCE on bacterial community

Soil microbes are the first to encounter chemicals entering the soil environment, which are sensitive indicators of soil contamination because they can rapidly respond to ecosystem disturbances (Nemir et al., 2010; Wu et al., 2019; Li et al., 2019b; Zhou et al., 2020, 2021). Microbial community structure can be dramatically impacted at about 1 ppm TCE concentration in soil (Nemir et al., 2010). Besides, TCE concentration above 10 mg/kg has been demonstrated can reduce soil quality, affect soil microbial biomass, and inhibit soil organic matter decomposition and mineral cycling (Li et al., 2019b). Furthermore, the downward movement of TCE along with soil depth affects the microbial ecology in different soil layers, and subsoil microbial diversity and functions were reduced compared to the topsoil (Koner et al., 2022). So far, only two articles have reported the effect of TCE concentration on soil microbial community, and there are few studies on the effects of different TCE concentrations on microbial communities in different types of soil.

In general, the entry of TCE into soil or groundwater ecosystem alters environmental quality, disrupts the natural balance of microbial communities, and affects microbial density and abundance, community structure, and metabolic activity (Fries et al., 1997; Nemir et al., 2010; Koner et al., 2022). Microbial density returned to baseline after subsequent phenol-TCE treatment in a field study, but the original species richness was not recovered until after toluene-TCE treatment (Fries et al., 1997). Additionally, TCE can change the composition of soil bacteria, fungi and actinomycetes in Mollisol, including *Acidobacteria, Proteobacteria*, *Planctomycetes*, *Chytridiomycota*, *Streptomycetales*, *Pseudonocardiales*, *Propionibacteriales*, and *Rhizobiales*, thereby affecting soil carbon and nitrogen cycling processes and energy metabolism (Li et al., 2019b). Their subsequent study manifested that TCE pollution had a significant effect on nitrogen transformation (Li et al., 2020). Nevertheless, the underlying mechanism of the effects of TCE on soil microbial community structure and function remains unclear.

TCE can influence the community structure and activity of methanotrophs, while the relative abundance of *Methylobacter* increased with TCE level and exposure time, suggesting that *Methylobacter* is resistant to TCE and plays a leading function in TCE degradation (Kong et al., 2014). Moreover, microorganisms with functions of TCE degradation have been reported, such as *Flavobacterium*, *Clostridium*, *Desulfotomaculum*, *Desulfuromonas*, *Nitrospira*, *Sphingomonas*, *Acidovorax*, *Bacillus*, and *Pseudomonas*, indicating the adaptability of native bacteria to TCE pollution (Koner et al., 2022). Thus, microorganisms can develop multiple physiological tolerance mechanisms and metabolic strategies under stressful conditions to degrade diverse environmental pollutants in ecosystems (Zhou et al., 2020, 2022; Koner et al., 2022). Studies have shown that many TCE-degrading microorganisms existed in TCE-contaminated sites (Lowe et al., 2002; de Guzman et al., 2018; Gafni et al., 2020). Reductive dechlorination bacteria have been proved to be closely related to the TCE-dechlorinating degradation in TCE-contaminated superfund sites (Lowe et al., 2002; de Guzman et al., 2018). Moreover, local microbial communities were reported can oxidize TCE in polluted water from Israeli Coastal Aquifer (Gafni et al., 2020). A recent study showed that the degree of soil contamination by TCE was positively correlated with the abundance of TCE-degrading taxa (Koner et al., 2022). Therefore, functional bacteria in TCE-contaminated environments are important microbial resources for TCE biodegradation.

## 3. Biodegradation of TCE

Biodegradation is one of the most promising technologies for TCE degradation in soil and groundwater, which involves four major processes: (1) anaerobic reductive dechlorination, an anaerobic process in which TCE is the electron acceptor, and hydrogen and organic substrate are as the electron donor (Pant and Pant, 2010; Tiehm and Schmidt, 2011); (2) anaerobic cometabolic reductive dichlorination, TCE can be cometabolized in the presence of growth-supporting electron acceptors (DCEs and VC; He et al., 2003; Tang et al., 2013; Clark et al., 2018); (3) aerobic co-metabolism, requiring oxygen for enzymatic degradation of TCE, and yields no good for related bacteria (Pant and Pant, 2010); (4) aerobic direct oxidation, aerobic bacteria can utilize TCE as the only carbon source, and the produced water and carbon dioxide are non-toxic to other residential microorganisms (Koner et al., 2022). The characteristics of the four main processes are shown in Table 2.

**Table 2:** Characteristics of four types of biodegradation process of TCE.

Reaction	Description	Characterization information	Toxic by-product	Half lives
Anaerobic reductive dechlorination	TCE is used as an electron acceptor, the bacteria may or may not gain energy by reduction of the compound. This reaction removes chloride atom from TCE and replaces it with a proton	Strictly anaerobic, requiring electron donors	Low chlorine substituted	Slow
Anaerobic cometabolic reductive dichlorination	TCE can be cometabolized in the presence of growth-supporting electron acceptors (DCEs and VC)	Strictly anaerobic, DCEs and VC as electron acceptors	Low chlorine substituted	Slow
Aerobic co-metabolism	TCE is fortuitously degraded by an enzyme used in cellular metabolism-typically monooxygenase and dioxygenase enzyme	Additional growth substrate	Epoxy compounds	Fast
Aerobic direct oxidation	Use of TCE as an electron donor for aerobic metabolism	Oxygen is needed	None	Fast

### 3.1. Anaerobic reductive dechlorination

#### 3.1.1. Bacterial isolates and mixed culture

In the anaerobic organohalide respiration, TCE acts as an electron acceptor, while chlorine is simultaneously removed from TCE (Ebrahimbabaie and Pichtel, 2021), and the energy from exergonic dehalogenation is utilized for microbial growth (Dolinova et al., 2017). In general, hydrogen and a variety of hydrogen-releasing substrates can be used as primary electron donors. The substrates are single compound substrates such as acetate, lactate, formate, pyruvate, benzoate, butyrate, methanol, ethanol, glucose, and propionate (Dolinova et al., 2017; Robles et al., 2021; Chen and Wu, 2022; Tomita et al., 2022; or complex substrates such as emulsified vegetable oil, soybean oil, surfactants, molasses, whey, and flour (Lee et al., 2012; Sheu et al., 2015; Dolinova et al., 2017; Chen and Wu, 2022; Dutta et al., 2022).

**Figure 2:**
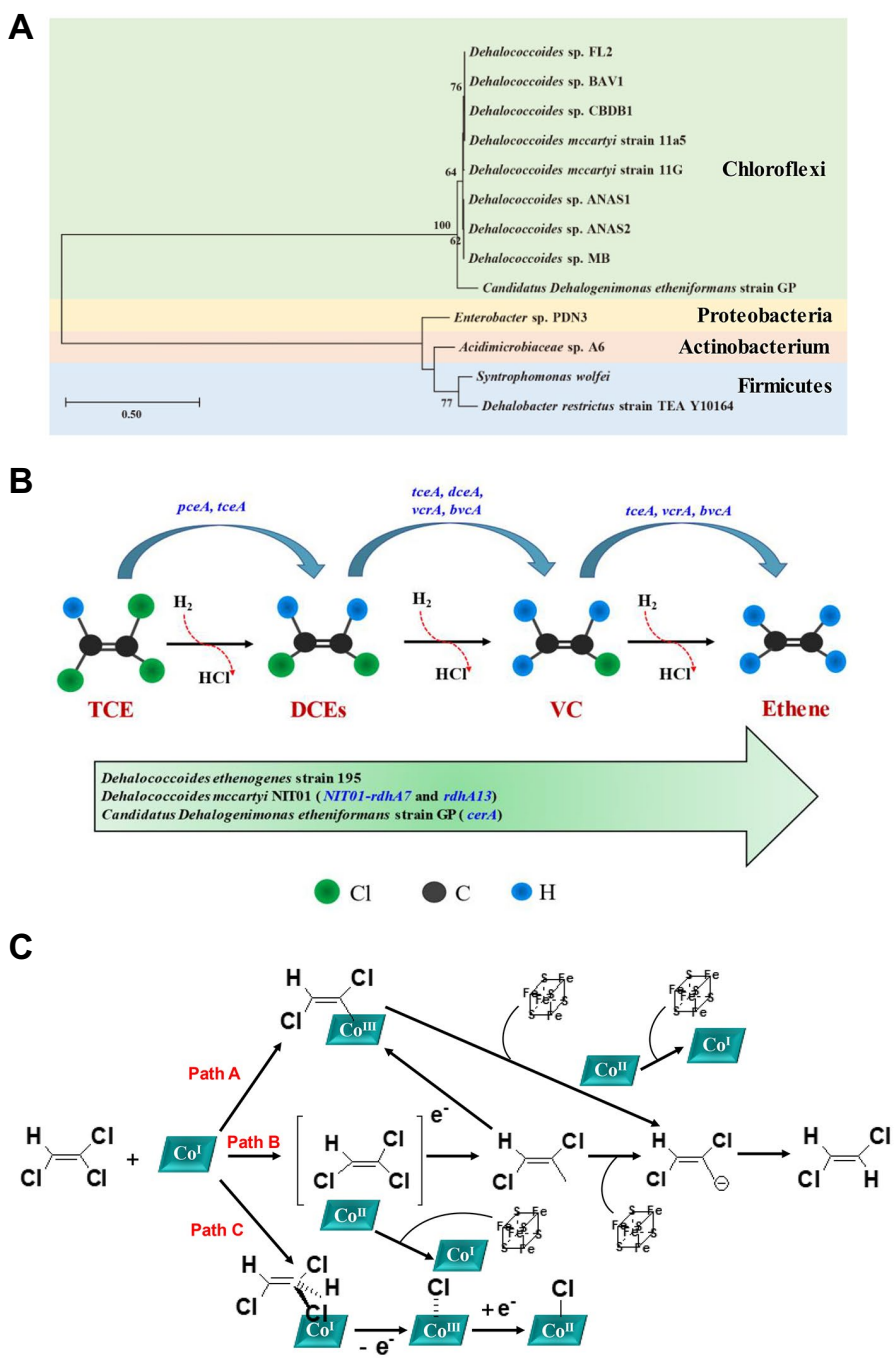
**(A)** Phylogenetic tree constructed based on the 16S rRNA sequences of bacterial isolates for anaerobic degradation of TCE; **(B)** Mechanism of completely anaerobic reductive dechlorination of TCE to ethene [adapted from (Maymo-Gatell et al., 1997; Dolinova et al., 2017; Lihl et al., 2019)]; **(C)** Proposed three reaction mechanisms of RDase: an organocobalt adduct (Path A), a single-electron transfer (Path B), and a halogen-cobalt bond (Path C; reproduced from (Jugder et al., 2015; Kunze et al., 2017).

Bacterial isolates and mixed culture involved in anaerobic reductive dechlorination of TCE were summarized in Table 3, and a phylogenetic tree was constructed with the identified bacterial isolates (Figure 2A). As for bacterial isolates, organohalide-respiring bacteria (OHRB) have been confirmed to be highly effective TCE degrading strains (Fincker and Spormann, 2017), including species from *Dehalococcoides* (Maymo-Gatell et al., 1997; Maymó-Gatell et al., 1999; Gushgari-Doyle and Alvarez-Cohen, 2020; Asai et al., 2022) and *Candidatus Dehalogenimonas* (Chen et al., 2022), which can dechlorinate TCE to benign ethene. Besides, strains from *Dehalobacter* (Holliger et al., 1998; Rupakula et al., 2015), *Enterobacter* (Kang et al., 2012), *Clostridium* (Lo et al., 2020; Lin et al., 2021), and *Acidimicrobiaceae* (Ge et al., 2019) were also observed to effectively dechlorinate TCE (Table 3). In addition, immobilization technique has been used to alleviate the toxic effect of TCE on cells (Lo et al., 2020), and the advantages including: (1) cells are well protected from adverse conditions, which can be easily recovered from bulk solutions; (2) cells can maintain high density for long time; (3) fixed cell can alleviate the inhibitory effects of high TCE concentrations to cells by avoiding delay times (Chen et al., 2007; Yang et al., 2019; Lo et al., 2020).

**Table 3:** Summary of anaerobic reductive dechlorination of TCE by bacterial isolates and mixed culture.

Bacterial isolates	Culture media	Inoculum	TCE concentration	Time	Dechlorination efficiency	References
*Dehalococcoides ethenogenes* 195	Basal salts medium	2% v/v	2.5 mmol/L	8 days	100%	Maymó-Gatell et al. (1999)
*Dehalococcoides* sp. strain FL2	–	–	–	–	–	He et al. (2005)
*Dehalococcoides* sp. strain GT	–	3% v/v	50 μmol/bottle	33 days	100%	Sung et al. (2006)
*Dehalococcoides* sp. strain MB	–	–	55 μmol/bottle	7 days	100%	Cheng and He (2009)
*Dehalococcoides* sp. strain CBDB1	Medium	30% v/v	45 mM	10 days	100%	Marco-Urrea et al. (2011)
*Dehalococcoides* strain ANAS1	–	–	–	–	–	Lee et al. (2011)
*Dehalococcoides* strain ANAS2	–	–	–	–	–	Lee et al. (2011)
*Dehalococcoides* strain BAV1	Biotrickling filter	200 ml (2 × 10^7^ cells/mL)	8–9 g m_bed_^−3^ h^−1^	40 days	45%	Popat et al. (2012)
*Dehalococcoides mccartyia* strain 11a	–	–	55 μmol/bottle	30 days	100%	Lee et al. (2013)
*Dehalococcoides mccartyi* strain 11a5	–	–	–	–	–	Lee et al. (2013)
*Dehalococcoides mccartyi* strain 11G	–	–	2.5 mM	40 days	100%	Zhao and He (2019)
*Dehalococcoides mccartyi* NIT01	Medium	–	4.0 mM	25 days	100%	Asai et al. (2022)
*Dehalobacter restrictus* strain TEA	–	–	–	–	–	Wild et al. (1996)
*Dehalobacter restrictus* PER-K23	–	–	–	–	–	Holliger et al. (1998)
*Candidatus Dehalogenimonas etheniformans* strain GP	–	–	–	–	100%	Chen et al. (2022)
*Enterobacter* sp. PDN3	Medium	OD660 = 1	72.4 μM	24 h	58%	Kang et al. (2012)
*Enterobacter* sp. PDN3	Medium	OD660 = 1	55.3 μM	5 days	80%	Kang et al. (2012)
*Clostridium* sp.	100 ml of synthetic groundwater, and 10 g of sediments	1–5% v/v	5 mg/L	100 days	up to 92%	Lin et al. (2021)
Immobilized *Clostridium butyricum* in silica gel	Microcosm (100 ml synthetic ground water and 10 g aquifer sediments)	5 ml inoculum in silica gels	2.5 mg/L	30 days	up to 69%	Lo et al. (2020)
*Acidimicrobiaceae* sp. A6	Column	10 ml	500 mg/L	21 days	22%	Ge et al. (2019)
**Mixed culture**	**Culture media**	**Inoculum**	**TCE concentration**	**Time**	**Dechlorination efficiency**	**References**
*Dehalococcoides* populations	Medium	4% v/v	0.13–0.2 mM	55 days	100%	Zhang et al. (2006)
*Dehalococcoides*-containing consortium (UC-1)	Minima medium	1% v/v	77.78 μmol/bottle	19 days	100%	Hu et al. (2011)
*Dehalococcoides mccartyi* 195 and *Syntrophomonas wolfei*	Medium	5% for each bacterium	78 μmol/bottle	18 days	88.4%	Mao et al. (2015)
Perchlorate reducing consortium	Medium	6% v/v	0.45 mmol/L	8 days	100%	Wen et al. (2017)

Regarding to mixed culture, *Dehalococcoides*-containing cultures have received more attention because they can effectively reduce TCE to ethene (Table 3; Freeborn et al., 2005; Zhang et al., 2006; Hu et al., 2011). TCE can be completely biodegraded through spatiotemporal changes in several dehalorespiring species, including *Sulfurospirillum*, *Dehalobacter*, *Desulfitobacterium*, *Geobacter*, and *Dehalococcoides* (Dugat-Bony et al., 2012). Furthermore, a novel perchlorate-reducing dechlorinating bacteria has been manifested can reduce TCE up to 0.45 mmol/L to nontoxic ethene within 8 days after two additions of TCE (Wen et al., 2017).

#### 3.1.2. Degradation mechanism, pathway, and enzyme

During reductive dechlorination, TCE acts as an electron acceptor in anaerobic microorganisms, linking the reductive dehalogenation of TCE with the synthesis of ATP through the electron transport chain (Wohlfarth and Diekert, 1997). Anaerobic microorganisms proliferate using H_2_ or organic substrates as electron donors, resulting in sequential substitution of chlorine atoms to produce lower chlorinated compounds (including cis-DCE, trans-DCE, VC), and eventually form ethene (Figure 2B; Dutta et al., 2022).

Reductive dehalogenase (RDase) is a key enzyme in OHRB such as *Dehalococcoides*, *Dehalobacter*, and is up-regulated during anaerobic reductive dechlorination (Zhang et al., 2020; Asai et al., 2022; Chen et al., 2022; Koner et al., 2022). RDase is a membrane-associated iron–sulfur protein containing an activated super nucleophilic form of the coenzyme vitamin B12, cob(I)alamin, which cleaves carbon-halogen bonds, and dechlorinates TCE *via* electroreduction (Zhang et al., 2020; Yan et al., 2021). The proposed three reaction mechanisms of RDase were presented in Figure 2C. The three reaction mechanisms were: (1) Path A: formation of a transient organocobalt adduct; (2) Path B: a single-electron transfer with Co^I^ being the initial electron donor; and (3) Path C: halogen-cobalt bond formation. Among these three mechanisms, Path B is a long-distance electron transfer in outer-sphere, which suggested that electrons were transferred from Co^I^ leading to substrate radical formation and finally to the formation of a carbanion after elimination of the halogen substituent. However, the internal mechanism as outer-sphere or inner-sphere route is still under debate. For example, Zhang et al. demonstrated that the B_12_-catalyzed reductive dechlorination of olefins in microbes should proceed through an inner-sphere electron transfer rather than outer-sphere electron transfer pathway (Zhang et al., 2021). Therefore, more efforts should be devoted to exploring the actual mechanisms of anaerobic reductive dechlorination. Overall, *pceA*, *tceA* (TCE to DCE), *tceA*, *dceA*, *vcrA*, *bvcA* (cis-DCE to VC), and *tceA*, *vcrA*, *bvcA* (VC to ethene) are crucial RDases involved in TCE reductive dechlorination process (Figure 2B; Ise et al., 2011; Popat and Deshusses, 2011; Dugat-Bony et al., 2012; Zinder, 2016; Dolinova et al., 2017; Mao et al., 2019; Yan et al., 2021; Rossi et al., 2022b). Furthermore, the recently discovered new strain *Dehalococcoides mccartyi* NIT01 contains 19 *rdhA* genes (including *NIT01*-*rdhA7* and *rdhA13*), which are basically the same as the encoded *vcrA* and *pceA* (Asai et al., 2022). Moreover, *Candidatus Dehalogenimonas etheniformans* strain GP can combine formate and H_2_ oxidation with the reduction of TCE, DCE isomers, and VC to benign ethene using acetate as the carbon source, and the *cerA* gene plays a crucial role (Chen et al., 2022).

#### 3.1.3. Influence factors

The reductive dechlorination of TCE is affected by biological aspects, physicochemical factors (e.g., pH, temperature, oxygen), and coexisting pollutants.

Co-culture of *Dehalococcoides mccartyi* 195 and *Syntrophomonas wolfei* can promote TCE degradation through efficient metabolic exchange and electron transfer as the result of interspecies aggregation during the syntrophic growth (Mao et al., 2015). The addition of *Shewanella oneidensis* MR-1 has been shown to have a positive effect on reductive dehalogenase activity and vitamin B12 uptake in cultures containing *Dehalococcoides*, which largely facilitated the complete dechlorination of TCE to ethene (Li et al., 2019c).

pH is a crucial factor affecting the growth of microbes (Yadvika et al., 2004). In an upflow anaerobic sludge blanket (UASB) reactor, TCE-degrading anaerobic granular sludge could effectively treated 36.5 mg/L TCE wastewater (80% removal rate) at pH 6.0–8.0 (Zhang et al., 2015a). Weakly acidic environment (pH < 7.0) has a greater influence on TCE removal (Zhang et al., 2015a). Temperature also plays a vital role in affecting the bioavailability of hydrophobic contaminants and influencing bacterial capability of growth and metabolism and enzymatic activities (Wang et al., 2022; Yamazaki et al., 2022). Temperature can influence the anaerobic degradation rate and products of TCE by a *Dehalococcoides*-containing consortium (UC-1), because it affects the acclimation period and leads to the selection of *Dehalococcoides* populations (Hu et al., 2013). Recently, Yamazaki et al. pointed out that the optimal temperature for TCE dechlorination in contaminated soil and groundwater is 25–35°C, and microbial community was notably impacted at 35°C (Yamazaki et al., 2022). In addition, oxygen can affect the dechlorination activity and change the overall biotransformation rate. The degradation of TCE by *Dehalococcoides mccarty* was prolonged when the oxygen concentration changed from 0 to 7.2 mg/L (Liu et al., 2017). Furthermore, hydraulic retention time affects the bacterial community structure, and TCE, as a recalcitrant compound, requires longer detention time for complete biodegradation. It was reported that the removal efficiency of TCE decreased from 99 to 85% when the hydraulic retention time is reduced from 25 to 5 h in a laboratory UASB reactor (Zhang et al., 2015b).

Many studies have shown that coexisting pollutants [e.g., chloroform, acetylene, perfluoroalkyl substances (PFASs), perfluoroalkyl acids (PFAAs), arsenic, perchlorate, and sulfate] can inhibit the biodegradation of TCE. Generally, the effects of these pollutants on the biodegradation of TCE are highly concentration dependent. Chloroform may affect dechlorinating organisms or may indirectly influence dechlorination by inhibiting other microorganisms (Duhamel et al., 2002). Chloroform in concentrations as low as 2.5 μM has been reported to inhibit TCE dechlorination by *Dehalococcoides* isolates and mixed cultures (Maymó-Gatell et al., 2001; Duhamel et al., 2002; Ding et al., 2020). Besides, acetylene influences a variety of microbial processes and typically inhibits redox-active metalloenzymes, which has been shown to be a potential inhibitor of a mixed anaerobic dehalogenation culture during TCE reductive dechlorination (Pon et al., 2003). The inhibition of acetylene on microbial processes is strongly dependent on its concentration. High concentration of acetylene (1.3 mM) reversibly inhibited TCE reductive dechlorination by *Dehalococcoides mccartyi* isolates and mixed cultures (Mao et al., 2017a). Fermentable components of Aqueous Film-Forming Foams (AFFFs) stimulated the dechlorination of TCE by microbial communities including *Dehalococcoides mccartyi*, while dechlorination could be inhibited by AFFF-derived perfluoroalkyl substances (PFASs; Harding-Marjanovic et al., 2016). Furthermore, the reductive dechlorination of TCE by a methanogenic mixed culture was significantly inhibited when exposed to perfluoroalkyl acids (PFAAs; 11 PFAA analytes, 6 mg/L each, totaling 66 mg/L; Weathers et al., 2016). Moreover, As(III) at a concentration of 9.1 μM reduced the cell growth of *Dehalococcoides mccartyi* by 50%, and affected the dechlorination activity of TCE (Gushgari-Doyle and Alvarez-Cohen, 2020). When the As(V) concentration reached 200 μM, there was no effect on TCE dechlorination at the initial stage, but inhibition was observed in cultures amended with 200 μM As(V) and 100 μM As(V) in 12 and 17 days, respectively, corresponding with the accumulation of As(III). Perchlorate preferentially uses electron donors compared to TCE, especially when electron donor is insufficient (Wen et al., 2016). Increasing perchlorate from 0 to 600 mg/L significantly decreased the relative abundance of TCE dechlorination bacteria *Dehalococcoides* (Wen et al., 2016). High sulfate concentration (5 mM) inhibited the reductive dechlorination of TCE by microbial communities containing *Dehalococcoides* due to the toxicity of the produced sulfide (Mao et al., 2017b).

### 3.2. Anaerobic cometabolic reductive dechlorination

Anaerobic cometabolic reductive dechlorination has also been described as a mechanism for anaerobic biodegradation of TCE (He et al., 2003; Tang et al., 2013; Clark et al., 2018). TCE can be co-metabolized in the presence of growth-supporting electron acceptors (He et al., 2003).

Cometabolic reductive dechlorination of TCE was first discovered in methanogenic cultures (Bouwer and McCarty, 1983; Vogel and McCarty, 1985). Methanogens and other bacteria have abundant reduced transition-metal cofactors that stochastically dechlorinate TCE under anaerobic conditions (Gantzer and Wackett, 1991). Unfortunately, the cometabolic reductive dechlorination rate of TCE decreases by an order of magnitude with each chlorine substituent removed, resulting in the accumulation of cis-DCE and VC (Gantzer and Wackett, 1991; Löffler et al., 2013). *Dehalococcoides mccartyi* strains play significant roles in catalyzing the reductive dechlorination of TCE to benign ethene under anoxic conditions (Magnuson et al., 2000; Loeffler et al., 2013; Clark et al., 2018). Notably, *bvcA*-carrying *Dehalococcoides mccartyi* strain BAV1 cannot grow with TCE, but can dechlorinate TCE to ethene when growth-supporting DCEs or VC are available (He et al., 2003; Krajmalnik-Brown et al., 2004; Löffler et al., 2013; Tang et al., 2013).

### 3.3. Aerobic co-metabolism

#### 3.3.1. Bacterial isolates and mixed culture

Aerobic co-metabolism is a significant and widely studied biodegradation process of TCE. TCE co-metabolism was first demonstrated by Wilson and Wilson that TCE can be aerobically co-metabolized to CO_2_ in unsaturated soil columns exposed to a mixture of natural gas in air (0.6%; Wilson and Wilson, 1985). Aerobic co-metabolic biodegradation of TCE can be achieved through the supplement of primary carbon sources (Arcangeli and Arvin, 1997), including ammonia; some aliphatic compounds, such as methane, ethane, propane, propene, butane, and isoprene; and aromatics, such as toluene, creosol, and phenol (Chang and Alvarez-Cohen, 1995; Chen et al., 2007; Shukla et al., 2014; Yang et al., 2019; Gafni et al., 2020; Table 4). Among these substrates, toluene is reported to be the most efficient and practicable substrate (Hunt et al., 1995; Arcangeli and Arvin, 1997).

**Table 4:** Aerobic co-metabolism of TCE by bacterial isolates and mixed culture.

Bacterial isolates	Culture media	Carbon source	TCE concentration	Time	Summary	References
*Pseudomonas cepacia* G4	Medium	Toluene	–	–	0.027–0.152 g of TCE/g of biomass	Landa et al. (1994)
*Pseudomonas cepacia* G4	M9 medium	Phenol	13.89 μM	–	7%	Sun and Wood (1996)
*Pseudomonas putida* Fl	M9 medium	Toluene	75 μM	6 h	71%	Sun and Wood (1996)
*Pseudomonas putida* Fl	Aqueous medium	L-arginine and toluene	36.5 mg/L	15 h	100%	Abu Hamed et al. (2013)
*Pseudomonas putida* Fl	Aqueous medium	L-arginine and toluene	55 mg/L	27 h	78%	Abu Hamed et al. (2013)
*Pseudomonas putida* Fl	Aqueous medium and 2-undecanone	L-arginine and toluene	55 mg/L	14 h	100%	Abu Hamed et al. (2013)
*Pseudomonas putida* Fl	M9 medium	Toluene	13.89 μM	–	7.15%	Sun and Wood (1996)
*Pseudomonas putida* B2	MSM	Toluene	20 mg/L	–	20%	Applegate et al. (1997)
*Pseudomonas putida* BCRC 14349	–	Phenol	0.2–20 mg/L	135 h	0.032 mg TCE/mg phenol	Chen et al. (2007)
*Pseudomonas stutzeri* OX1	–	Toluene	100 μM	15 h	25, 28%	Ryoo et al. (2001)
*Pseudomonas stutzeri* OX1	–	Glucose	100 μM	15 h	19%	Ryoo et al. (2001)
*Pseudomonas Mendocina* KR1	M9 medium	Toluene	13.89 μM	–	11.74%	Sun and Wood (1996)
*Pseudomonas* sp. strain ASA86	Medium	Toluene and tryptophan	1 mg/L	18 h	50%	Chee (2011)
*Pseudomonas fluorescens*	Medium	Phenol	0.1 mg/L	3 days	80%	Li et al. (2014)
*Pseudomonas plecoglossicida*	Soil slurry	Toluene	10 mg/kg	9 days	81.70%	Li et al. (2015)
*Mycobacterium vaccae* JOB5	Basal media	Propane	20 μM	24 h	removal up to 99% TCE	Wackett et al. (1989)
*Mycobacterium vaccue*	–	Propane	37.5 μM	72 h	53%	Vanderberg et al. (1995)
*Burkholderia cepacia* G4	Medium	Toluene	–	3 weeks	65%	Mars et al. (1996)
*Burkholderia* sp. strain TAM17	Medium	Toluene and tryptophan	1 mg/L	7 days	50%	Chee (2011)
*Comamonas testosteroni* strain R5	Medium	Phenol	3.8 μM	73 h	100%	Futamata et al. (2001)
*Comamonas testosteroni* RF2	MSM	Phenol	122.5 μg/L	5 days	100%	Zalesak et al. (2017)
*Methylosinus trichosporium* OB3b	MSM	–	About 80 μM	1 h	TCE removal: 56%	Tsien et al. (1989)
*Methylomonas*	Phosphate buffer	Methane, phenol or toluene	0.5 mg/L	8 days	0.01–0.13 mg of	Hanada et al. (1998)
					TCE/mg of dry cell weight	
*Alcaligenes eutrophus* JMP134	Medium	–	20 μM	14 h	40–60%	Harker and Kim (1990)
*Xanthobacter* sp. Strain Py2	Potassium phosphate buffer	Propane	–	–	0.03–0.34 g of TCE/g of biomass	Reij et al. (1995)
Immobilized *Rhodococcus* sp. L4	MSM	Toluene	2–68 μM	–	–	Suttinun et al. (2010)
*Cupriavidus* sp. CY-1	Medium	Phenol	5–50 mg/L	96 h	74–85%	Chang et al. (2021)
**Mixed culture**	**Culture media**	**Carbon source**	**TCE concentration**	**Time**	**Summary**	**References**
Methane oxidizing bacteria	Unsaturated soil	Natural gas (0.6%)	150 μg/L	2 weeks	TCE removal: 99%	Wilson and Wilson (1985)
Methane-utilizing mixed culture CL-M	Medium	Methane	7.5 ppb	20 h	100%	Fogel et al. (1986)
*Pseudomonas* and *Methylosinus trichosporium* OB3b	M9 medium	Toluene	75 μM	6 h	93–99%	Sun and Wood (1996)
Consortium of five bacterial strains (*Pseudomonas putida, Pseudomonas fluorescens, Mycobacterium* sp., *Nocardia paraffinae,* and *Nitrosomonas europeae*)	Medium	Toluene	228–2,500 mg/L	1 week	16–47 μg TCE/L/h	Meza et al. (2003)
Toluene-oxidizing bacteria (*Ralstonia* sp. P-10 and *Pseudomonas putida*)	Slurry microcosms	Toluene	546 ± 69 μg/L	1 days	62%	Han et al. (2007)
Toluene-oxidizing bacteria (*Ralstonia* sp. P-10 and *Pseudomonas putida*)	*In-situ* pilot	Toluene	430 ± 119 μg/L	3 days	>90%	Han et al. (2007)
Consortium included *Mycobacterium* sp., *Pseudomonas fluorescens*, *Pseudomonas putida* and *Nocardia paraffinae*	Medium	–	1,682 mg/L	1 week	89.67%	Cutright and Meza (2007)
Consortium composed of *Comamonas testosteroni* RF2 and	MSM	Phenol and lactate sodium	116 μg/L	6 days	Nearly 100%	Zalesak et al. (2021)
*Mycobacterium aurum* L1						

**Figure 3:**
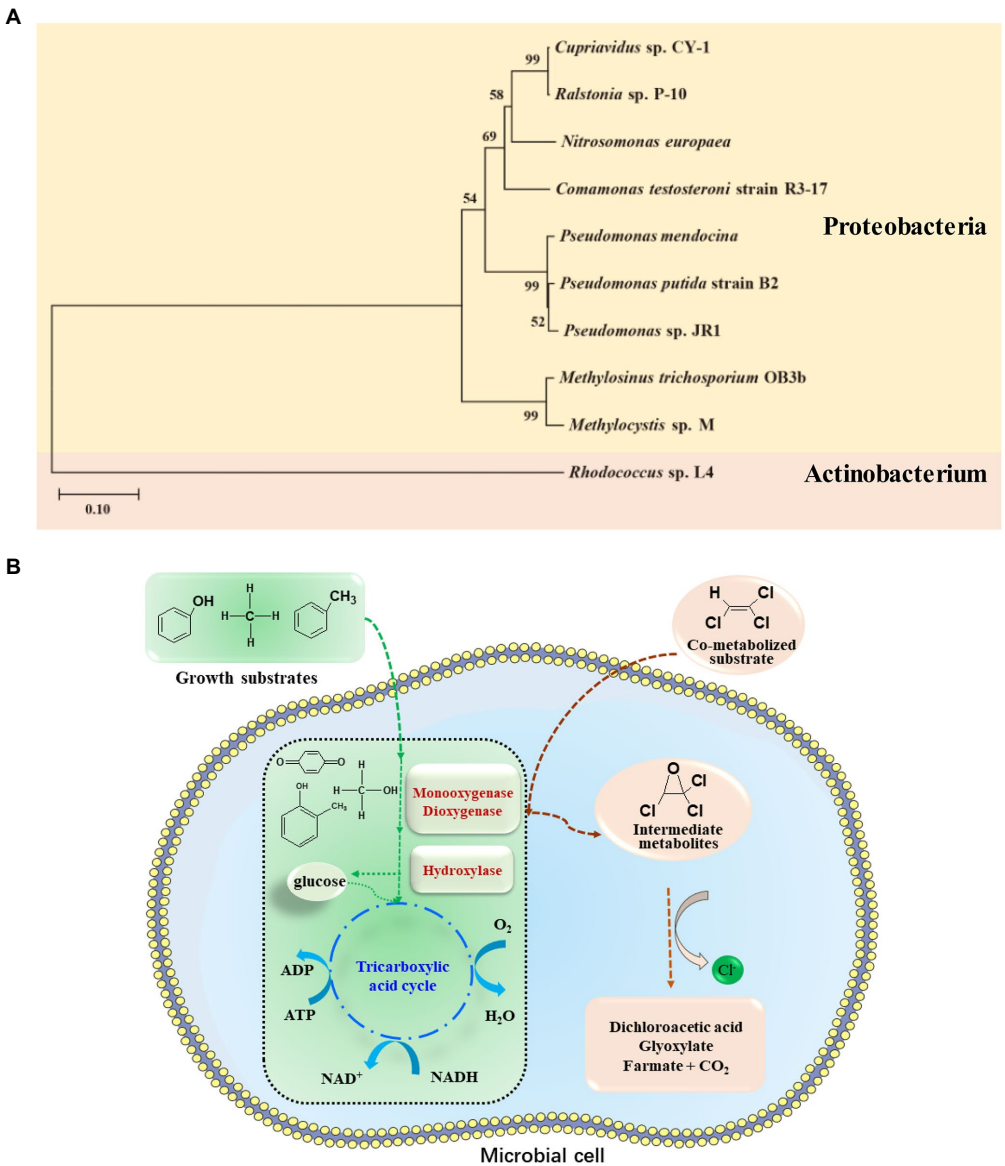
**(A)** Phylogenetic tree constructed based on the 16S rRNA sequences of isolated bacteria for TCE aerobic co-metabolism degradation; **(B)** Conceptual diagram of mechanism of aerobic co-metabolism of TCE with toluene, methane, and phenol.

Bacterial isolates and mixed cultures that can aerobic co-metabolic degradation of TCE were displayed in Table 4. The bacterial phylogenetic tree was constructed based on the 16S rRNA sequences of isolated TCE biodegradation bacteria (Figure 3A). Most *Pseudomonas* sp. strains exhibit strong resistance to organic solvents, and some of them can aerobically co-metabolize TCE in the presence of toluene and phenol (Winter et al., 1989; Landa et al., 1994; Sun and Wood, 1996; Applegate et al., 1997; Ryoo et al., 2001; Chen et al., 2007; Chee, 2011; Abu Hamed et al., 2013; Li et al., 2014, 2015). Moreover, other strains from *Mycobacterium* (Wackett et al., 1989; Vanderberg et al., 1995), *Burkholderia* (Mars et al., 1996; Chee, 2011), *Comamonas* (Futamata et al., 2001; Zalesak et al., 2017), *Methylosinus* (Tsien et al., 1989), *Methylomonas* (Hanada et al., 1998), *Alcaligenes* (Harker and Kim, 1990), *Xanthobacter* (Reij et al., 1995), and *Cupriavidus* (Chang et al., 2021) were also effective TCE co-metabolizing bacteria (Table 4). Immobilized strain has also been proved to be an important method for promoting co-metabolism of TCE (Chen et al., 2007; Yang et al., 2019).

As for mixed cultures, methane oxidizing bacteria (Wilson and Wilson, 1985; Fogel et al., 1986; Hanada et al., 1998), toluene-oxidizing bacteria (Han et al., 2007), phenol-degrading *Variovorax* strains (Futamata et al., 2005), as well as consortium bacteria including species from *Pseudomonas, Mycobacterium, Methylocystis*, *Methylosinus*, *Nocardia*, *Nitrosomonas*, *Bordetella*, *Burkholderia*, *Ralstonia*, and *Comamonas* have also been demonstrated to be important mixed cultures for TCE aerobic co-metabolism (Table 4; Sun and Wood, 1996; Hanada et al., 1998; Meza et al., 2003; Cutright and Meza, 2007; Zalesak et al., 2021).

Based on the above-described bacterial isolates and mixed cultures, single-stage, dual-stage, and three-stage reactors were designed for co-metabolize of TCE. Single-stage reactor systems of biofilm and immobilized strains were proposed for the co-metabolic degradation of TCE (Fitch et al., 1996; Arcangeli and Arvin, 1997; Shimomura et al., 1997; Yang et al., 2019). Besides, dual-stage reactor system was adopted to provide primary substrate and oxygen for cell growth in one compartment and send cells to the substrate-free compartment to provide TCE to avoid competitive inhibition during TCE degradation (Tschantz et al., 1995). In a two-stage bioreactor operating in single-pass and crossflow, >78 and 93% TCE degradation rates were achieved for 20 and 10 mg/L of TCE by *Methylosinus trichosporium* OB3b (Tschantz et al., 1995). Furthermore, a laboratory-scale three-stage rotating biological contactor was used to treat TCE-containing synthetic wastewater with a mixed biofilm populations including nitrifiers, heterotrophs, and *Thiosphaera pantotropha*, which achieved almost 100% removal rate when the TCE load was 0.0039 m^3^/m^2^ day (Brar and Gupta, 2000).

#### 3.3.2. Degradation mechanism, pathway, and enzyme

Co-metabolism or co-metabolic degradation typically describes the ability of a microorganism to convert a non-growth supporting substrate (co-metabolized substrate or co-substrate) in the presence of a growth supporting substrate (primary substrate; Suttinun et al., 2013). Thus, the primary substrate supports microbial growth, while TCE does not enter the catabolic and anabolic pathways of microbial cell (Figure 3B). There are three reasons to elucidate why co-substrates such as TCE do not support microbial growth (Suttinun et al., 2013): (1) initiating enzymes transform substrates to products that are not further converted by other enzymes in the microorganism to generate metabolic intermediates for biosynthesis and energy production; (2) the initial substrate is converted to products that hinder subsequent enzyme activity during mineralization or inhibit bacterial growth; and (3) microorganisms need different substrates to trigger specific responses.

During aerobic co-metabolism, TCE binds to the enzymatic active site of various physiological substrates and is oxidized. As shown in Table 5, TCE can be oxidized by oxygenase generating microorganisms with broad auxiliary substrates, including methane, toluene, butane, ammonia, etc. Oxidation of TCE by monooxygenase, dioxygenase, or hydroxylase can lead to the formation of TCE epoxides, which are unstable and can be nonenzymatically degraded to various products, including glyoxylate, dichloroacetate, formate, and carbon dioxide (Figure 3B; Arp, 1995). Typically, methane oxidizing bacteria (methanotrophs) can use methane as sole carbon source, and co-metabolize TCE through nonspecific methane monooxygenase (MMO) enzymes (Shukla et al., 2009). In this process, MMO catalyzes the transformation of methane to methanol, and ultimately co-metabolizes TCE. Two genetically unrelated MMOs have been found and observed to be regulated by copper concentration (Shukla et al., 2009; Semrau et al., 2010), including soluble MMO (sMMO) expressed only by a subset of methanotrophs and ubiquitous membrane-bound particulate MMO (pMMO). The sMMO has the advantage of degrading TCE (Shukla et al., 2009), and purified pMMO can mineralize TCE to CO_2_ (Lontoh et al., 2000). Regarding toluene oxidizers, toluene monooxygenase and dioxygenase are efficient functional genes for TCE degradation, widely distributed in *Pseudomonas* (Table 5). In addition, isopropyl benzene dioxygenase (Dabrock et al., 1992; Pflugmacher et al., 1996), butane monooxygenase (Halsey et al., 2005), alkene monooxygenase (Ensign et al., 1992; Saeki et al., 1999), ammonia monooxygenase (Arciero et al., 1989; Rasche et al., 1991; Kocamemi and Cecen, 2010), phenol hydroxylase, and dichlorophenol hydroxylase (Harker and Kim, 1990) play crucial roles in the co-metabolic degradation of TCE (Table 5).

**Table 5:** Physiological substrates, bacteria, and enzymes involved in TCE co-oxidation.

Physiological substrate	Representative bacteria	Enzyme	References
Methane	*Methylosinus trichosporium* OB3b	sMMO	Fitch et al. (1996); Fox et al. (1990); Jahng and Wood (1994); Oldenhuis et al. (1989); Sullivan et al. (1998); Tsien et al. (1989)
——	*Methylococcus capsulatus*	pMMO	Lontoh et al. (2000)
Methane	*Methylocystis* sp. M	MMO	Kikuchi et al. (2002); McDonald et al. (1997); Shimomura et al. (1997)
Methane	Methanotrophic mixed culture	pMMO	Anderson and McCarthy (1997)
Methane	Methanotrophic consortia	MMO	Wang et al. (2019)
Methane	*Methylocella* sp.	pMMO and sMMO	Shao et al. (2019)
		(*pmoA* and *mmoX* genes)	
Methane	*Methylocystis* community	pMMO and sMMO (*pmoA* and *mmoX* genes)	Shukla et al. (2009)
Toluene	*Pseudomonas cepacia* G4	Toluene ortho-monooxygenase	Landa et al. (1994); Shields et al. (1991)
Toluene	*Pseudomonas mendocina*	Toluene para-monooxygenase	Winter et al. (1989)
Toluene	*Pseudomonas putida* F1	Toluene dioxygenase	Liu et al. (2012); Nelson et al. (1988)
–	*Pseudomonas putida*	Toluene dioxygenase	Nelson et al. (1988)
–	*Pseudomonas*	Toluene oxygenase	Lee et al. (2008)
Toluene and glucose	*Pseudomonas stutzeri* OX1	Toluene-o-xylene monooxygenase	Ryoo et al. (2001)
Toluene and tryptophan	*Pseudomonas* sp. strain ASA86	*todC1* gene product encoding toluene dioxygenase	Chee (2011)
Isopropyl benzene	*Rhodococcus erythropolis* BD2	Isopropyl benzene dioxygenase	Dabrock et al. (1992)
Isopropyl benzene	*Pseudomonas* sp. strain JR1	Isopropyl benzene dioxygenase	Pflugmacher et al. (1996)
Butane	*Nocardioides* sp. CF8	Butane monooxygenase	Halsey et al. (2005)
Butane	*Pseudomonas butanovora*	Soluble butane monooxygenase	Halsey et al. (2005)
Propylene	*Xanthobacter*	Propylene (or alkene) monooxygenase	Ensign et al. (1992)
Propene	*Rhodococcus corallinus*	Alkene monooxygenase	Saeki et al. (1999)
Ammonia	*Nitrosomonas europaea*	Ammonia monooxygenase	Arciero et al. (1989); Kocamemi and Cecen (2010); Rasche et al. (1991)
Phenol and 2,4-dichlorophenoxyacetic acid	*Alcaligenes eutrophus* JMP134	Monooxygenase: phenol hydroxylase and dichlorophenol hydroxylase	Harker and Kim (1990)

#### 3.3.3. Influence factors

Studies have demonstrated that salinity, organic loading rate, toluene concentration, surfactant, and metal ion concentration are important influence factors affecting the aerobic co-metabolism of TCE. NaCl is a noncompetitive inhibitor for the degradation of toluene and TCE. As the instantaneous salinity increased from 0 to 3.5%, the maximum degradation rate of TCE by toluene-oxidizing cultures decreased from 2.28 to 1.45/day (Lee and Liu, 2006). In a methanogenic-methanotrophic coupled reactor, TCE degradation was influenced by organic loading rates, and possibly mediated by the effect of organic loading rate on methane (Lyew and Guiot, 2003). Increasing of organic loading rate was associated with increased dissolved methane level, which will lead to increased competition with TCE for the MMO and decreased TCE degradation (Lyew and Guiot, 2003). In a continuously fed biofilm reactor, TCE degradation by a toluene-oxidizing biofilm was strongly inhibited when toluene concentration exceeded 1 mg/L (Arcangeli and Arvin, 1997). Furthermore, biodegradable surfactants (Simple Green™ and soya lecithin) and primary substrate (cane molasses) can be used by indigenous microorganisms to improve the biodegradation efficiency of TCE during the aerobic co-metabolic process (Liang et al., 2011). Notably, metal ion concentrations have been shown to affect the co-metabolic biodegradation of TCE. Copper concentration can regulate the relative expression of pMMO, which has been proved to be a significant factor in the oxidation of TCE by *Methylobacter* sp. strain BB5.1 (Smith et al., 1997). Furthermore, 20 mg/L iron or 1 mg/L nickel enhanced the degradation of toluene and TCE by immobilized *Pseudomonas putida* F1 (Yang et al., 2019). This may be due to nickel and iron can provide necessary cofactors for the enzyme and improve the removal efficiency. A recent study found that the addition of low concentrations of zinc and copper facilitated the enzymatic conversion process and bioremediation of TCE in water by *Pseudomonas plecoglossicida* (Qiu et al., 2022).

### 3.4. Aerobic direct oxidation

Aerobic direct oxidation of TCE is a promising biological technology because it does not require auxiliary substrates and all available oxygen can be directly used for biodegradation (Gaza et al., 2019; Xing et al., 2022). In direct oxidation, microorganisms acquire organic carbon and energy by oxidatively degrading TCE (Pant and Pant, 2010). Mineralization of total TCE by aerobic microorganisms requires 2 moles of oxygen per mole of TCE, as shown in Eq. (1; Sun et al., 1998). As shown in Eq. (2), the oxidative biodegradation of TCE releases CO_2_ and H_2_O as end products (Baskaran and Rajamanickam, 2019).


(1) $$C_{2}{\mathrm{HCl}}_{3}+\mathrm{NADH}+H^{+}+2O_{2}\to 2{\mathrm{CO}}_{2}+{\mathrm{NAD}}^{+}+3\mathrm{HCl}$$

(2) $$C_{2}{\mathrm{HCl}}_{3}+O_{2}\overset{Microorganism}{\to }{\mathrm{CO}}_{2}+H_{2}O+\mathrm{Heat}+\mathrm{Biomass}$$

**Figure 4:**
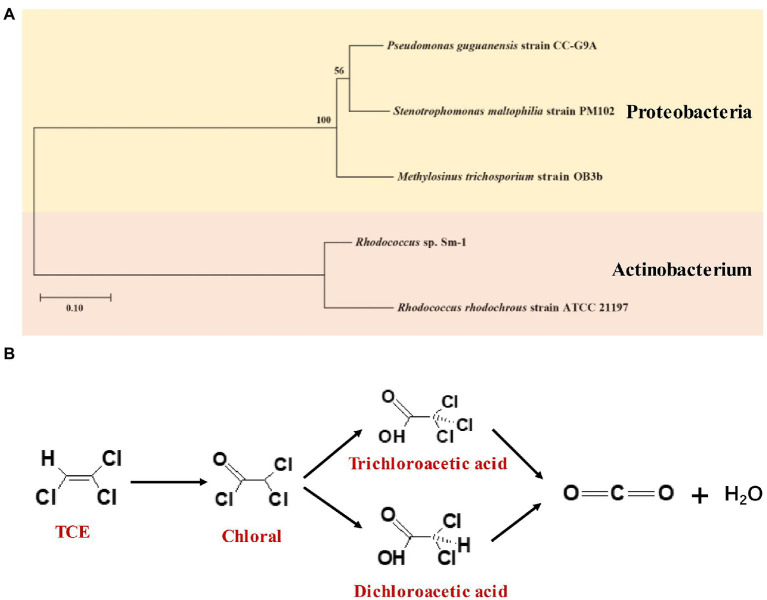
**(A)** Phylogenetic tree constructed based on the 16S rRNA sequences of isolated bacteria for TCE aerobic direct oxidation; **(B)** Aerobic direct oxidation of TCE by *Pseudomonas guguanensis* NR135725 [adapted from (Baskaran and Rajamanickam, 2019)].

Bacterial isolates and mixed cultures using TCE as the sole growth substrate were summarized in Table 6. The phylogenetic tree of the bacterial isolates was presented in Figure 4A. *Rhodococcus* sp. strains Sm-1 and *Rhodococcus rhodochrous* were able to mineralize 85 and 89% of TCE (1.1 mg/L) within 14 days, respectively (Malachowsky et al., 1994). The mineralization efficiencies of TCE (75 μM) by *Pseudomonas* and *Methylosinus* ranged from 71 to 109% in 6 h (Sun and Wood, 1996). Both two studies illuminated that oxygenase expression was responsible for TCE biodegradation (Malachowsky et al., 1994; Sun and Wood, 1996). In addition, *Stenotrophomonas maltophilia* PM102 degraded 90% of TCE (1.28 g/L) in 48 h at pH 7 and 77% of TCE in 72 h at pH 5 (Mukherjee and Roy, 2012). In a batch reactor, the removal rate of TCE (300 mg/L) reached >90% by microorganisms from turkey litter compost, among which *Pseudomonas guguanensis* NR135725 was the predominant strain for degrading TCE to CO_2_ and H_2_O (Baskaran and Rajamanickam, 2019). The proposed metabolic pathway of TCE aerobic biodegradation by *Pseudomonas guguanensis* NR135725 is shown in Figure 4B. A bacterial culture SF has been shown to degrade up to 400 mM TCE with an optimal temperature at 22°C and pH 7 in fixed-bed reactors and in batch experiments (Gaza et al., 2019).

**Table 6:** Aerobic direct oxidation of TCE by bacterial isolates and mixed culture.

Bacterial isolates	Media	Inoculum	TCE concentration	Time	Summary	References
*Rhodococcus* sp. strain Sm-1	Phosphate-buffered medium	–	1.1 mg/L	14 days	85%	Malachowsky et al. (1994)
*Rhodococcus rhodochrous*	Phosphate-buffered medium	–	1.1 mg/L	14 days	89%	Malachowsky et al. (1994)
*Pseudomonas cepacian* G4 PR1	M9 medium	5 ml (8%) OD600 = 1	75 μM	6 h	77 ± 6%	Sun and Wood (1996)
*Pseudomonas cepacian* G4	M9 medium	5 ml (8%) OD600 = 1	75 μM	6 h	62 ± 1%	Sun and Wood (1996)
*Pseudomonas mendocina* PR1	M9 medium	5 ml (8%) OD600 = 1	75 μM	6 h	85 ± 1%	Sun and Wood (1996)
*Pseudomonas putida* F1	M9 medium	5 ml (8%) OD600 = 1	75 μM	6 h	51 ± 3%	Sun and Wood (1996)
*Methylosinus trichosporium* OB3b	M9 medium	5 ml (8%) OD600 = 1	75 μM	6 h	109 ± 4%	Sun and Wood (1996)
*Pseudomonas guguanensis* NR135725	MSM	–	300 mg/L	–	93%	Baskaran and Rajamanickam (2019)
Immobilized *Bacillus* sp.	–	–	–	–	–	Dey and Roy (2009)
*Stenotrophomonas malto-philia* PM102	Chloride free minimal medium (pH 7)	–	1.28 g/L	48 h	90%	Mukherjee and Roy (2012)
*Stenotrophomonas malto-philia* PM102	Chloride free minimal medium (pH 5)	–	1.28 g/L	72 h	77%	Mukherjee and Roy (2012)
**Mixed culture**	**Media**	**Inoculum**	**TCE concentration**	**Time**	**Summary**	**References**
Consortium microorganism from turkey litter compost	MSM	4% OD600 = 1.1	300 mg/L	55 h	>90%	Baskaran and Rajamanickam (2019)
Mixed bacterial enrichment culture (SF culture)	Fixed-bed reactors and batch experiments	–	400 μM	–	100%	Gaza et al. (2019)
*Comamonas testosteroni* RF2 and *Mycobacterium aurum* L1	MSM	–	115.7 μg/L	21 days	100%	Zalesak et al. (2021)
A nitrifying bacterial consortium	Medium	1 ml (20%) OD600 = 0.232	500 mg/L	144 h	58.63 and 62.7%	Berrelleza-Valdez et al. (2019)

Compared with anaerobic biodegradation and aerobic co-metabolism, the aerobic direct oxidation process of TCE has the advantage of no by-products. Therefore, it is necessary to isolate more aerobic bacteria for direct oxidation degradation of TCE in the future, and the mechanism of aerobic direct oxidation should be further studied.

### 3.5. Combined anaerobic and aerobic biodegradation

A combination of anaerobic (reduction) and aerobic (oxidation) is usually required to achieve TCE mineralization rather than partial conversion (Tartakovsky et al., 2003; Tresse et al., 2005; Dolinova et al., 2017). In particular, sequential anaerobic/aerobic biodegradation can conquer the shortcomings of using anaerobic and aerobic biodegradation alone (Bhatt et al., 2007; Tiehm and Schmidt, 2011; Frascari et al., 2013), and has the following main advantages: (1) prevents the accumulation of toxic metabolites; (2) does not require highly sensitive bacteria of the genus *Dehalococcoides*; (3) requires fewer electron donors as auxiliary substrates (Tiehm and Schmidt, 2011). So far, limited researches are available on the coupling of aerobic and anaerobic biodegradation of TCE (Tartakovsky et al., 2003; Tresse et al., 2005; Frascari et al., 2013; Polasko et al., 2019). A mutualistic consortium (especially methanotrophic and methanogenic microorganisms) almost completely degraded TCE at a loading of 18 mg/L_R_/day through the combination of reduction and oxidation pathways in an ethanol-fed biofilm reactor (Tartakovsky et al., 2003). Furthermore, TCE was completely degraded by different microorganisms using a three-step sequence of aerobic/anaerobic/aerobic treatment in batch bioreactors (Frascari et al., 2013).

## 4. Material-mediated enhanced biodegradation

**Figure 5:**
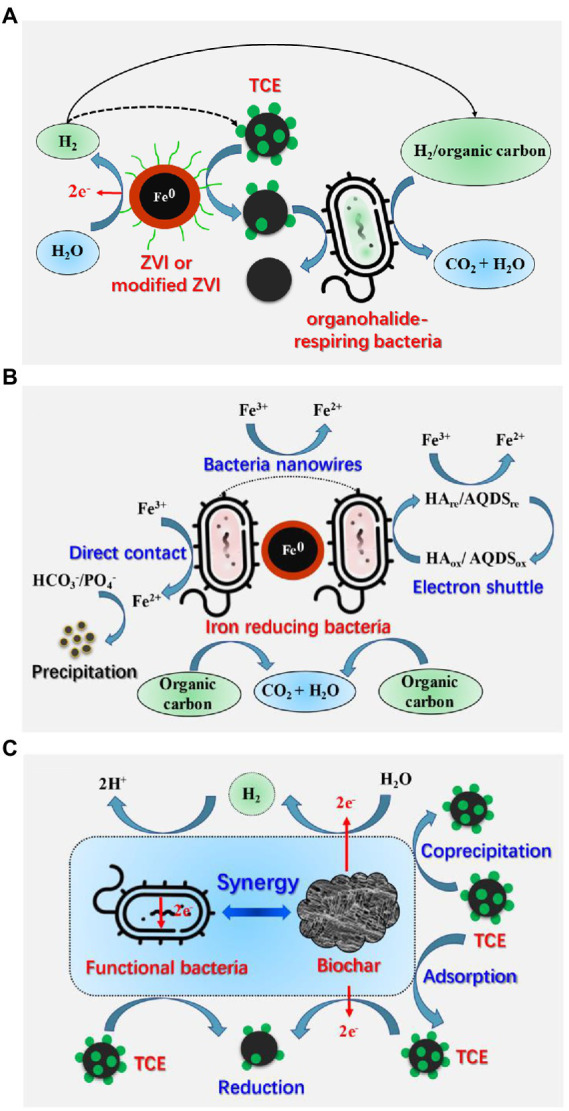
Mechanisms of material-mediated enhanced biodegradation of TCE. **(A)** The coupling mechanism of organohalide-respiring bacteria with ZVI or modified ZVI (reproduced from (Dong et al., 2019); **(B)** The coupling mechanism of iron reducing bacteria with ZVI [reproduced from (Dong et al., 2019)]; **(C)** The coupling mechanism of microorganisms with biochar.

Biodegradation of TCE takes a long time, and tends to result in the accumulation of regulated and more toxic transformation products, such as VC (Bruton et al., 2015). Therefore, more studies adopt enhanced biodegradation technology, especially material-mediated combined technologies to improve the biodegradation of TCE. From the existing researches, ZVI and biochar combined with microorganism have received more attention (Figure 5). Combined physicochemical and biological systems are more efficient for TCE degradation (Wang and Tseng, 2009; Wang et al., 2016).

### 4.1. ZVI-microorganism

Microbial reductive dechlorination combined with ZVI can improve TCE removal in several ways (Xiu et al., 2010; Tian and Yu, 2020; Wang et al., 2020): (1) ZVI can rapidly remove high concentration of TCE at early stage (Chen et al., 2012); (2) ZVI can reduce redox potential and enhance microbial activity of some functional bacteria (Chen et al., 2012; Wang et al., 2016, 2020); (3) H_2_ produced by ZVI is safer than the liquid hydrogen stored in steel tanks (Chen et al., 2012), which can be used as electron donor for anaerobic bacteria, including OHRB and iron reducing bacteria (IRB; Wang and Tseng, 2009; Xiu et al., 2010; Wang et al., 2016; Dong et al., 2019); (4) iron is an essential trace element for enzymes and catabolism and anabolism of microorganisms (Tian and Yu, 2020); (5) the low-valent iron Fe(II) and Fe(0) can be used as electron donors for microbes, while Fe(III) can be utilized as terminal electron acceptor for IRB such as *Shewanella* and *Geobacter* (Tian and Yu, 2020).

Many researches have been devoted to investigate the synergetic effects of ZVI, mZVI, nZVI, nZVI-CMC, FeS and functional microorganisms in promoting TCE removal (Figure 5A; Wang and Tseng, 2009; Kocur et al., 2016; Wang et al., 2016, 2020; Yang et al., 2018; Tian and Yu, 2020; Wu et al., 2020; Li et al., 2021; Yuan et al., 2022). The ZVI-bacteria combined system is not only a cost-effective technology, but also can greatly improve the TCE removal rate (Wang and Tseng, 2009). Sulfate reducing bacteria-ZVI system enables *in situ* sulfidation of ZVI, which offers a valuable strategy to overcome the limitations of biological or abiotic dechlorination degradation for TCE at sites (Islam et al., 2021). As for mZVI, the removal efficiency of TCE by mZVI combined with AHB was higher than that of pure mZVI, and particle sizes and dosages of the mZVI showed significant effects on the remediation performance of the mZVI-AHB system (Yuan et al., 2022). Furthermore, a field study suggested that mZVI combined with biostimulation can form a neutral, anoxic, and reducing condition in groundwater, which removed approximately 97.5% of chlorinated ethylene (including 12.8 and 14.23 μg/L TCE) and 80.2% of chlorinated ethane in 253 days (Wu et al., 2020). As for nZVI, *in situ* injection of nZVI stabilized with carboxymethyl cellulose (nZVI-CMC) significantly increased the functional bacterial populations (*Dehalococcoides*) and vinyl chloride reductase (*vcrA*) genes, thus promoting the complete and long-term dechlorination of chlorinated ethenes (Kocur et al., 2015, 2016). Moreover, co-encapsulated nZVI and *Dehalococcoides* species BAV1 system can degrade 10 mg/L of TCE within 3 h (Shanbhogue et al., 2017). Additionally, FeS has been demonstrated can enhance electron transfer for TCE dechlorination by *Dehalococcoides mccartyi* strain 195 (Li et al., 2021).

IRB can reduce Fe(III) to Fe(II) through multi-pathways and remove corrosion products on ZVI surface, thereby prolonging the service life of ZVI and improving the degradation efficiency of target pollutants (Figure 5B; Yang et al., 2017; Dong et al., 2019; Li et al., 2019a). For example, the combined system of ZVI and *Shewanella alga* BrY was able to degrade about 50% of TCE (30 mg/L) in 25 days (Shin et al., 2007), and the combination of *Shewanella putrefaciens* with aged mZVI can degrade 30 mg/L of TCE to 13 mg/L in about 20 days (Yang et al., 2017). Both studies revealed that Fe(II) ions generated by microbial reduction of Fe(III) could efficiently improve the TCE removal. However, nZVI may inhibit the removal rate of TCE by model IRB *Shewanella algae* CCM 4595, because the adsorption of cells to the passivated iron surface hinders electron transfer (Honetschlägerová et al., 2018).

### 4.2. Biochar-microorganism

Biochar-microorganism collaboration can promote the biodegradation of TCE (Figure 5C; Liu et al., 2021; Rossi et al., 2022b). The advantages include: (1) the adsorption of TCE on biochar can create a low-toxic environment for microorganisms; (2) the multi-layer porous structure of biochar can provide a habitat for microorganisms; (3) biochar can promote the biodegradation of TCE by stimulating the metabolic activity of functional anaerobic microorganisms (Aktaş et al., 2012; Liu et al., 2021). The biochar and landfill leachate microorganism packing column system can remove up to 99.7% of TCE (35 mg/L) removal, and the biochar prepared from waste material can support the formation of dechlorinated biofilms and facilitate the bioremediation of TCE (Siggins et al., 2021). Recently, commercial polyhydroxy butyrate combined with pine biochar improved the bioreductive dechlorination of TCE (approximately 80 mg/day) from aqueous solutions in a new reactor setup and small pilot scale (Rossi et al., 2022a). In a subsequent study, they confirmed that biofilm-biochar reactor was able to remove more than 99% of 100 μM TCE, and that *Dehalococcoides mccartyi* and *tceA*, *bvcA*, and *vcrA* genes played significant roles in TCE biodegradation (Rossi et al., 2022b). Furthermore, the addition of biochar supported nanoscale iron sulfide composite (CMC-FeS@biochar) followed by *Corynebacterium variabile* HRJ4 achieved 99% of TCE (10 mg/L) removal, and acetylene was the main product of the chemical process, whereas ethylene was the main product of the biological process (Lyu et al., 2018).

## 5. From laboratory scale studies to field application

According to the USEPA, bioremediation accounts for 24% of soil and groundwater remediation technologies (Wang et al., 2022). Compared with energy-intensive physical and chemical treatment methods, *in situ* bioremediation is an efficient method for TCE removal with less environmental impact and lower energy consumption (Rao et al., 2022; Rossi et al., 2022a; Yamazaki et al., 2022). Generally, three different approaches have been used widely for *in situ* bioremediation of TCE (Figure 1; Dolinova et al., 2017): (1) monitored natural attenuation (MNA), which uses natural abiotic and biotic degradation processes, and monitors the TCE plume over time to control the rate of natural attenuation and achieve site-specific remediation goals in time; (2) biostimulation, which combines the principle of MNA but involves the addition of carbon sources or electron donors to support and enhance TCE degradation by native microbial populations in contaminated environments; (3) bioaugmentation, which refers to the addition of pre-cultured degrading bacteria when the functional bacteria in the target environment are insufficient, and successful bioaugmentation often requires the addition of electron donors. Over the past few decades, numerous efforts have been dedicated to improve TCE biodegradation through biostimulation, bioaugmentation, and a combination of biostimulation and bioaugmentation (Löffler and Edwards, 2006; Polasko et al., 2019; Lo et al., 2020; Zalesak et al., 2021; Dutta et al., 2022; Underwood et al., 2022). It is worth noting that most of the field applications were conducted in the United States.

### 5.1. Biostimulation

The addition of single-compound (including phenol, toluene, nitrogen, phosphorus, methane, lactate) and complex substrates (such as slow polycolloid-releasing substrate, molasses, nZVI-CMC) has been shown to significantly promote the *in situ* TCE bioremediation. At the Moffett site (formerly the Moffett Naval Air Station), Mountain View, CA, phenol and toluene were used as the main substrates for the biodegradation of TCE, achieving 90% removal of 250 μg/L TCE in groundwater (Hopkins and McCarty, 1995). Delivery of vapor toluene with air promoted the growth of native toluene-utilizing bacteria that can degrade TCE through aerobic co-metabolism, and finally degraded TCE in aquifers to less than 10 mg/L (>90% removal; Kuo et al., 2004). Nitrogen and phosphorus addition has been observed can increase methanotroph populations and improve TCE biodegradation, which can influence at least 60 ft. above and to each side of the horizontal injection well at the Savannah River Area M bioremediation site, USA (Brockman et al., 1995). Besides, the addition of triethyl-phosphate and nitrous oxide to the pulsed injection of methane significantly stimulated *in situ* TCE degradation in groundwater at Westinghouse Savannah River Site in Aiken, South Carolina (Palumbo et al., 1995). A subsequent study at the same site confirmed that the injection of nutrients (methane, nitrogen, and phosphorus) facilitated the growth of TCE-degrading microorganisms and the biodegradation of TCE (Pfiffner et al., 1997). What’s more, injection of methane as an electron donor into the Snake River aquifer beneath the Test Area North site of the Idaho National Laboratory stimulated reductive dechlorination and co-metabolism of TCE by microorganisms in groundwater (Conrad et al., 2010). Complete biodegradation of TCE was achieved by lactate biostimulation of spatiotemporal changes in dehalorespiring bacterial community at a chlorinated solvent-contaminated site in France (Dugat-Bony et al., 2012).

For complex substrates, slow polycolloid-releasing substrate containing vegetable oil, cane molasses, and surfactants has been successfully adopted to remediate TCE contaminated groundwater in an industrial park site located in southern Taiwan (Tsai et al., 2014; Kao et al., 2016), and it removed up to 99% of TCE (1872 μg/L) in 50 days (Tsai et al., 2014). Another study in a TCE-contaminated groundwater site in Taiwan demonstrated that 97% of TCE was effectively degraded within 600 days with molasses injections (Liu et al., 2020). The above studies generally assumed that the existence of certain functional bacteria (e.g., *Dehalococcoides*, *Dehalogenimonas*, *Pseudomonas*, *Sulfuricurvum*) and genes (e.g., *tceA*, *vcrA*) were responsible for TCE biodegradation (Tsai et al., 2014; Kao et al., 2016; Liu et al., 2020). In addition, injection of nZVI-CMC stimulated natural OHRB and promoted dechlorination of chlorinated ethenes to nontoxic ethene at a field site in Sarnia, Ontario (Kocur et al., 2015, 2016). In the nZVI-CMC injection system, nZVI can generate hydrogen to create strong reducing conditions for bioreductive dechlorination, and at the same time, CMC is rapidly metabolized into cellulose, which is beneficial to *in situ* microbial reductive dechlorination (Kocur et al., 2015).

### 5.2. Bioaugmentation

Bioaugmentation refers to the direct injection of selected exogenous organisms into contaminated areas to facilitate the biodegradation of pollutants (Steffan et al., 1999). *Methylosinus trichosporium* OB3b and *Burkholderia cepacian* ENV435 have been manifested as two effective bacteria for field application (Duba et al., 1996; Steffan et al., 1999). Additionally, bioaugmentation using a mixed dechlorination culture KB-1 has been reported to significantly enhance *in situ* anaerobic bioremediation of TCE-contaminated groundwater (~13°C) in fractured carbonate rock at a site in Southern Ontario, Canada (Perez-de-Mora et al., 2014). Interestingly, a 3 to 4 orders of magnitude increase in the number of *Bacteroidetes* population in KB-1 was associated with an increase in ethene conversion (Perez-de-Mora et al., 2014). Recently, in a TCE-contaminated fractured rock aquifer, Trenton, N.J. in the USA, a bioaugmentation test with an emulsified vegetable oil solution (EOS®) and a dechlorinating consortium (KB-1®; containing *Dehalococcoides*) suggested that ethene levels were correlated strongly with *Candidatus Colwellbacteria* (*p* < 0.05), emphasizing the importance of functional bacterial populations for *in situ* biodegradation of TCE (Underwood et al., 2022).

### 5.3. Combination of biostimulation and bioaugmentation

So far, there are only two studies on the combination of biostimulation and bioaugmentation to degrade TCE. *Dehalococcoides*-containing cultures have been used in combined biostimulation and bioaugmentation systems. A pilot test at Launch Complex 34, Cape Canaveral Air Force Center indicated that native microbial communities can dechlorinate TCE to ethene when supplied with electron donors (methanol, ethanol, acetate, and lactate); however, bioaugmentation by a dechlorinating culture KB-1 markedly promoted the formation rate of ethene, and the removal rate of biological treatment was over 98.5% (Hood et al., 2008). They found that the abundance of *Dehalococcoides* increased by 2 orders of magnitude after biostimulation and bioaugmentation. A study at a TCE-contaminated groundwater site in Ft. Lewis, WA in the USA reported that biostimulation with whey (consisting of 10–13% of protein and 70–75% of lactose) and bioaugmentation with a *Dehalococcoides*-containing culture was an effective strategy to enhance TCE dechlorination by altering microbial populations (Lee et al., 2012).

## 6. Challenges and future perspectives

Based on the above review and discussion, several challenges and perspectives should be considered by future research:

Although some TCE-degrading microorganisms have been isolated and described, so far, the number of strains that can be applied *in situ* and completely dechlorinate TCE is very limited. For example, some effective *Dehalococcoides* strains are highly sensitive bacteria, which may restrict their use in field conditions. Therefore, it is necessary to isolate or discover more tolerant and efficient strains, such as facultative bacteria, to improve the efficient and complete biodegradation of TCE in laboratory and field applications. More importantly, compared with pure culture, the isolation and enrichment of highly efficient mixed cultures for TCE biodegradation is an important research direction.

In addition, the degradation intermediate products and metabolic mechanisms of TCE by different genera are different, and more TCE degradation pathways and genes need to be fully elucidated in the future. The coexistence of various microorganisms, such as the growth of other native bacteria (e.g., methanogenic, sulfate-reducing bacteria), can compete with *Dehalococcoides* strains for electron donors in the subsurface environment. Hence, the dynamic and long-term interactions between different microorganisms and their effects on TCE removal should be investigated thoroughly.

Currently, the genetic mechanisms associated with the anaerobic and aerobic degradation of TCE remain unclear. The integration of genomics, metagenomics, proteomics, and systems biology is a powerful tool to disclose novel bacterial functional genes of TCE degradation bacteria and provide useful information for further environmental bioremediation. Furthermore, advanced molecular biology techniques (such as compound-specific isotope analysis, fluorescence *in situ* hybridization, qPCR, and stable isotope probing) need to be developed and applied to evaluate the *in situ* biodegradation processes of TCE.

Long-term biodegradation of TCE can lead to accumulation of toxic products. Therefore, a combination of physicochemical and microbial degradation is necessary to promote the complete dehalogenation of TCE. For example, material- or nanomaterial-mediated enhanced biodegradation is a promising technique. Furthermore, the underlying mechanisms of the combined and enhanced biodegradation should be investigated comprehensively. Besides, more green and sustainable technologies need to be developed for TCE *in situ* remediation. Since natural attenuation is a low-cost and highly sustainable technology that integrates a series of naturally occurring biological, physical, and chemical processes (biodegradation, adsorption, volatilization, etc.), it should be widely adopted in field-scale studies.

A few anaerobic and aerobic bacterial isolates and mixed cultures have been reported to degrade TCE under laboratory conditions by batch, microcosmic experiments, and bioreactors. Further *in situ* studies (especially studies combining biostimulation and bioaugmentation) are needed to evaluate the biodegradation efficiency of functional microorganisms in field applications. It is worth noting that most of the field application was done in the USA, mainly around the 2000s. Therefore, current research should pay more attention to field studies and developing countries.

## 7. Conclusion

Biodegradation serves as a great promise in dealing with TCE pollution. Anaerobic reductive dechlorination, anaerobic cometabolic reductive dichlorination, aerobic co-metabolism, and aerobic direct metabolism are the four major biodegradation processes leading to TCE biodegradation in bacterial isolates and mixed cultures. Enzymes play crucial roles in TCE biodegradation, such as RDase for anaerobic reductive dechlorination, and monooxygenase, dioxygenase, and hydroxylase for aerobic co-metabolism. The combination of anaerobic and aerobic biodegradation is a safe and effective method to complete TCE biodegradation. The combination of microorganisms with ZVI, modified ZVI, or biochar is an effective material-mediated TCE biodegradation technology. In addition, the current research on the biodegradation of TCE in field application mainly focuses on biostimulation, bioaugmentation, and the combination of biostimulation and bioaugmentation. Finally, the challenges and prospects of TCE biodegradation are proposed based on current research. We hope this review can provide specific guidance and recommendations for future laboratory and field studies of CAHs biodegradation.

## Author contributions

ZW: conceived and designed the manuscript, drafted the manuscript, reviewed and polished the article, and funding acquisition. QM: methodology, software, and reviewed and polished the article. HN, HS, and RL: methodology and reviewed and polished the article. HL: methodology, reviewed and polished the article, and funding acquisition. GR: reviewed and polished the article and funding acquisition. FZ, CP, and BL: reviewed and polished the article. XM: designed the manuscript, reviewed and polished the article, and funding acquisition. All authors contributed to the article and approved the submitted version.

## Funding

The authors gratefully acknowledge financial support for this work from the Ministry of Science and Technology of the People’s Republic of China (2020YFC1808603), the National Natural Science Foundation of China (22106037, 42177218, 21876042, and 22106036), and the S&T Program of Hebei (21374204D and 22374206D).

## Conflict of interest

The authors declare that the research was conducted in the absence of any commercial or financial relationships that could be construed as a potential conflict of interest.

## Publisher’s note

All claims expressed in this article are solely those of the authors and do not necessarily represent those of their affiliated organizations, or those of the publisher, the editors and the reviewers. Any product that may be evaluated in this article, or claim that may be made by its manufacturer, is not guaranteed or endorsed by the publisher.

## Figures and Tables

**Table:** 

TCE	Trichloroethylene
CAH	Chlorinated aliphatic hydrocarbon
DNAPL	Dense non-aqueous phase liquid
cis-DCE	Cis-1,2-dichloroethylene
tans-DCE	Trans-1,2-dichloroethene
DCE	1,1-Dichloroethene
VC	Vinyl chloride
MNA	Monitored natural attenuation
UASB reactor	Upflow anaerobic sludge blanket reactor
OHRB	Organohalide-respiring bacteria
RDase	Reductive dehalogenase
MMO	Methane monooxygenase
sMMO	Soluble methane monooxygenase
pMMO	Particulate methane monooxygenase
IRB	Iron reducing bacteria
ZVI	Zero-valent iron
nZVI	Nanoscale zero-valent iron
mZVI	Microscale zero-valent iron
nZVI-CMC	nZVI stabilized with carboxymethyl cellulose
MSM	Mineral salts medium
